# *MT1DP* loaded by folate-modified liposomes sensitizes erastin-induced ferroptosis via regulating miR-365a-3p/NRF2 axis in non-small cell lung cancer cells

**DOI:** 10.1038/s41419-020-02939-3

**Published:** 2020-09-14

**Authors:** Chengcheng Gai, Chuanliang Liu, Xinghan Wu, Mengyu Yu, Jie Zheng, Weifen Zhang, Shijun Lv, Wentong Li

**Affiliations:** 1 Department of Pathology, Weifang Medical University, Weifang, Shandong Province 261014 China; 2 The Second Department of Health Care, Weifang People’s Hospital, Weifang, Shandong Province 261041 China; 3 Department of Pharmacology, Weifang Medical University, Weifang, Shandong Province 261014 China

**Keywords:** Cancer, Cell biology

## Abstract

Although ferroptosis has been recognized as a novel antitumoral treatment, high expression of nuclear factor erythroid 2-related factor 2 (NRF2) has been reported to be an antioxidant transcript factor that protects malignant cells from ferroptosis. Previous findings indicated that metallothionein 1D pseudogene (*MT1DP*), a long noncoding RNA (lncRNA), functioned to aggravate oxidative stress by repressing antioxidation. Here we aimed at assessing whether *MT1DP* could regulate erastin-induced ferroptosis on non-small cell lung cancer (NSCLC) and elucidating the mechanism. We found that ectopic expression of *MT1DP* sensitized A549 and H1299 cells to erastin-induced ferroptosis through downregulation of NRF2; in addition, ectopic *MT1DP* upregulated malondialdehyde (MDA) and reactive oxygen species (ROS) levels, increased intracellular ferrous iron concentration, and reduced glutathione (GSH) levels in cancer cells exposed to erastin, whereas downregulation of *MT1DP* showed the opposite effect. RNA pulldown assay and dual-luciferase reporter assay confirmed that *MT1DP* modulated the expression of NRF2 via stabilizing miR-365a-3p. As low solubility of erastin limits its efficient application, we further prepared folate (FA)-modified liposome (FA-LP) nanoparticles for targeted co-delivery of erastin and *MT1DP* to enhance the bioavailability and the efficiency of the drug/gene combination. Erastin/*MT1DP*@FA-LPs (E/M@FA-LPs) sensitized erastin-induced ferroptosis with decreased cellular GSH levels and elevated lipid ROS. In vivo analysis showed that E/M@FA-LPs had a favorable therapeutic effect on lung cancer xenografts. In short, our findings identify a novel strategy to elevate erastin-induced ferroptosis in NSCLCs acting through the *MT1DP*/miR-365a-3p/NRF2 axis.

## Introduction

Ferroptosis, which is a novel subtype of cell death different from other types of cell death both morphologically and biochemically^1,2^, is characterized by depletion of glutathione (GSH) and overgeneration of lipid reactive oxygen species (ROS) depending upon intracellular iron ions^1,3^.

Erastin has been identified as a prototype of recently discovered Ras-selective ferroptotic compounds^4^; however, previous studies confirmed that a variety of tumor cells were insensitive to erastin^5^. nuclear factor erythroid 2-related factor 2 (NRF2) has been reported to be an important transcription factor that protects malignant cells from oxidative stress, chemotherapeutic agents, and facilitates cancer progression^6,7^. Suppression of NRF2 contributed to an increased oxidative stress level and accelerated ferroptosis^8,9^.

A growing number of evidences has suggested that some long noncoding RNAs (lncRNAs) are involved in cancer initiation and progression. Recently, lncRNAs have been found to act as part of the cellular antioxidant system that orchestrates signaling pathways to fine-tune cell survival and death in response to external stresses^10,11^. Previous findings indicated that an lncRNA, metallothionein 1D pseudogene (*MT1DP*), functioned to aggravate oxidative stress by repressing NRF2-mediated antioxidation^12,13^.

Here we confirmed that *MT1DP* attenuated expression of NRF2 and increased sensitivity of NRF2-overexpressed non-small cell lung cancer (NSCLC) cells to erastin-induced ferroptosis via stabilizing miR-365a-3p. We demonstrated that the cell viability of NSCLC cell lines was significantly restrained by erastin and exogenous *MT1DP* accompanied with augmented lipid peroxidation. We further developed a nano-delivery system based on folate-modified liposomes (FA-LP) to co-delivery *MT1DP* and erastin(E/M@FA-LPs). In vitro and in vivo experiments indicated that E/M@FA-LPs displayed strong killing effects on tumor cells.

## Material and methods

### Cell culture

*KEAP1* mutant NSCLC cell lines (A549) and *KEAP1* non-mutant NSCLC cell lines (H1299) were acquired from American Type Culture Collection (Manassas, VA, USA). A549, H1299 were cultured in RPMI-1640 supplemented with 10% fetal bovine serum (FBS; Invitrogen, CA, USA). All cell lines were maintained at 37 °C and 5% CO_2_ in a humid environment.

### Tissue samples

A total of 64 cases of human NSCLC tissue samples (32 cases with mutant *KEAP1*, 32 cases with non-mutant *KEAP1*) from November 2013 to October 2017 were pathologically diagnosed at the Affiliated Hospital of Weifang Medical University. Prior informed consent for the use of tissue samples was obtained from all patients and the study was approved by the Ethics Committees of the Weifang Medical University.

### Bioinformatics analysis

Ferroptosis-inducer data for differential genetic analysis were obtained from NCBI publicly available genomics database, Gene Expression Omnibus (GEO) DataSets (GSE94550, GSE137952, and GSE122985). The R software package was used to process and identify the differential expressed genes.

### Preparation of LPs for co-loading of erastin and *MT1DP*

Precisely weighed 120 mg of l-α-phosphatidylcholine (Solarbio, Beijing, China), 40 mg of cholesterol (Solarbio), 20 mg of DSPE-PEG-FA (Shanghai Ponsure Biotechnology, China, Shanghai), and 3 mg of erastin (Sigma-Aldrich, MO, USA) were fully dissolved in 5 mL chloroform and mixed with 3 mL of 1 mg/mL erastin chloroform solution. Chloroform was then removed using a rotary evaporator to form a dry-lipid film at 35 °C under uniform speed. *MT1DP* (300 nM) dissolved in phosphate-buffered saline (PBS) (pH 7.4, 5 mL) was added to the lipid film and hydrated for 60 min. After the hydration was completed, the cells were sonicated with an ultrasonic cell disruptor and passed through a polyethersulfone membrane with a membrane pore diameter of 450 and 220 nm to decrease the particle size.

### Loading capacity, encapsulation efficiency, and drug-release studies

The E/M@FA-LPs suspension was filtered through a 0.22 µm microfilter membrane, the quantity of erastin in the supernatant was analyzed using the reverse-phase high-performance liquid chromatography (HPLC) analysis method. encapsulation efficiency (EE %) and loading capacity (LC %) were calculated using equations as previously described^14^. The quantity of erastin was measured using the Shimadzu Prominence HPLC system and C18 analytic column (Luna C18(2) 25 cm × 4.6 cm, 5 mm, Phenomenex, Inc., Torrance, CA). The ultraviolet absorbance was measured at a wavelength of 275 nm and their cumulative release was calculated.

### Plasmid construction and cell transfection

MiR-365 mimic (5′-UAAUGCCCCUAAAAAUCCUUAU-3′) and miR-365 inhibitor (5′-AUAAGGACCCCCAGGGGCAUUA-3′) were chemically synthesized by Sangon (Shanghai, China). *MT1DP* was subcloned into pEGFP-N1 vector as previously described^15^. *MT1DP* short hairpin RNA (shRNA) and NRF2 shRNA sequences were ligated into the pGPU6/GFP/Neo vector to construct si*MT1DP* and siNRF2 plasmids. pcDNA3-EGFP-C4-NRF2 plasmid^16^ was acquired from Addgene (Catalog: 21549; Cambridge, MA, USA). The overexpression plasmids and interference plasmids were extracted by QIAGEN Plasmid Extraction Kit. The mixture containing RNA-overexpressing or -interfering plasmid was added to Lipofectamine 2000 (Invitrogen, Carlsbad, CA, USA) and incubated in NSCLC cells.

### Cell viability assay

Cells (1 × 10^3^/well) were seeded in a 96-well plate and treated with or without erastin (10 µM/mL) for 48 h. MTT (3-(4,5-dimethylthiazol-2-yl)-2,5-diphenyltetrazolium bromide, 5 mg/mL) solution (10 µL/well) was added into the medium and incubated for 4 h; MTT solution was discarded and 100 µL dimethyl sulfoxide was added. The absorbance was measured at 490 nm by SpectraMax M5 plate reader (Molecular Devices, Sunnyvale, CA, USA).

### EdU proliferation assay

To measure cell proliferation, 5-ethynyl-2′-deoxyuridine (EdU) proliferation assay was performed. Cells were plated in 24-well plates at a density of 5 × 10^4^ cells/well. Twenty-four hours later, cells were treated with 10 µM EdU (RiboBio, Guangzhou, China) and fixed with 4% paraformaldehyde. The sections were imaged using a fluorescence microscope and the number of proliferating cells was averaged to calculate the labeling index.

### Wound-healing and transwell invasion assay

Cells/well (1 × 10^5^) were plated into a six-well plate and incubated for 24 h and a straight line was scratched across the surface of cells. After incubation for 24 h, migration distance was calculated. Transwell invasion assay was performed using Millipore transwell chambers (8 μm pore size; Millipore, Billerica, MA, USA). A549 cell (2 × 10^4^ cells/well) transfected with *MT1DP* were seeded in the top chamber in 100 μL serum-free medium. The lower chambers were filled with 600 μL complete medium with 10% FBS. After 24 h incubation, 0.1% crystal violet dye was used to stain cells. The images were analyzed by NIH ImageJ software (National Institutes of Health, Bethesda, MD, USA).

### ROS assay and lipid peroxidation assessment, and measurement of intracellular GSH

Intracellular ROS was measured using dichlorofluorescein diacetate fluorescent probe detection kit according to the manufacturer’s instruction (Thermo Fisher Scientific, Waltham, MA, USA), results were monitored with fluorescence microscope or SpectraMax M5 plate reader. Malondialdehyde (MDA) assay kit was used to assess lipid peroxidation according to the manufacturer’s instruction; for the measurement of intracellular GSH, *MT1DP* overexpression cells were treated with or without erastin for 24 h, cells were sonicated, and the supernatant was used to detect intracellular GSH by GSH assay kit (Beyotime Biotechnology, Nanjing, China). GSH content was expressed as a ratio to the absorbance, which was measured at 412 nm.

### Ferrous iron assay

Cells were plated at 1 × 10^5^ cells/well in a six-well plate; 24 h later, they were treated with erastin and/or NRF2 inhibitor. The intracellular ferrous iron level was assessed with an iron colorimetric assay kit (Abcam, Cambridge, UK) according to the manufacturer’s instruction.

### Flow cytometry

Cells/well (1 × 10^5^; A549 or H1299 cells) were plated in a six-well plate; 24 h later, *MT1DP*-overexpressing cells were treated or not with erastin. Cells were suspended in 100 µL binding buffer and stained with Annexin V-fluorescein isothiocyanate (FITC) and 7-Aminoactinomycin (Annexin V-FITC Kit; Beckman Coulter, Marseille, France) according to the manufacturer’s instruction. Cell ferroptosis was analyzed using flow cytometry (FCM; FACSCalibur, BD Biosciences, Franklin Lakes, NJ, USA).

### Transmission electron microscope assay

For analyzing the morphology of mitochondria, 2 × 10^5^ cells/well were seeded in 6-well plate and exposed to erastin for 12 h. After that, cells were collected, washed three times with PBS, and fixed with 2.5% glutaraldehyde. Samples were then pretreated according to standard procedures including staining, dehydration, embedding, and slicing to obtain ultra-thin sections. During the analysis, images were acquired using a HITACHIH-7000 transmission electron microscope (TEM; Hitachi, Tokyo, Japan).

### Mitochondrial membrane potential (ΔψM) assay

Fluorescent dye 5, 5′, 6, 6′-tetrachloro-1,1′,3,3′-tetraethyl benzimidazolyl-carbocyanineio-dide (JC-1, Beyotime Biotech, Nanjing, China) was used to monitor ΔψM. Cells were seeded in a six-well plate at 3 × 10^5^ cell/well. Loss of ΔψM was determined by decrease in JC-1 aggregates (red) and increase in JC-1 monomers (green). Cells were visualized by laser scanning confocal microscope (Leica TCS SP8, Solms, Germany). The excitation wavelength is 488 nm and the emission wavelengths of the JC-1 monomeric form and JC-1 aggregate form were 529 and 590 nm, respectively. The images were analyzed by NIH ImageJ software.

### Dual-luciferase reporter assay

The 3′-untranslated region (UTR) fragment of NRF2 mRNA containing the potential binding site of miR-365a-3p was inserted into a pMIR-REPORTTM luciferase reporter vector (Ambion, Austin, TX, USA) and the resulting plasmid was named NRF2-3′-UTR-WT. Another vector containing a mutant NRF2-3′-UTR at the binding site of miR-365a-3p was constructed and named NRF2-3′-UTR-MUT. Luciferase activity was measured in the form of chemiluminescence using the dual-luciferase reporter assay system (Promega, Madison, MI, USA) according the manufacturer’s instruction.

### RNA pulldown assay

A biotinylated DNA probe, similar to the sequence of miRNA-365, was synthesized and dissolved in 500 mL wash/binding buffer (0.5 M NaCl, 20 mM Tris-HCl, and 1 mM EDTA). The probe was incubated within cell lysates from A549 and H1299 cells (1 × 10^7^), then streptavidin-coated magnetic beads (Sigma) were added for 2 h at 25 °C. The *MT1DP* present in the pulldown complex was detected by quantitative reverse transcriptase PCR analysis.

### Western blot analysis

Cell were lysed using NP-40 lysis buffer (Roche, Basel, Switzerland) and the extracted proteins were separated by SDS-polyacrylamide gel electrophoresis and transferred onto polyvinylidene difluoride transfer membrane (Merck Millipore, Billerica, USA). The membrane was incubated with the appropriate anti-NRF2 and anti-GAPDH antibodies (Abcam, Cambridge, UK) overnight at 4 °C. The blots were developed with a peroxidase-conjugated secondary antibody and the proteins were visualized using by ECL Plus Detection Reagent (Merck Millipore, Billerica, USA). The gray-scale value was assessed by Gel-Pro analyzer.

### Quantitative real-time PCR

Total RNA was isolated using Trizol (Invitrogen, CA, USA) and first-strand complementary DNA was synthesized using the Reverse Transcription System Kit according to the manufacturer’s instructions (Takara Bio, Shiga, Japan). Complementary DNA from various cell samples was amplified with specific primers. NRF2: 5′-CACATCCAGTCAGAAACCAGTGG-3′ and 5′-GGAATGTCTGCGCCAAAAGCTG-3′; *MT1DP*: 5′-TCAAGGCCAAAGGTGGCTCCTGCAC-3′, and 5′-GCACGGCAGCTGCACTTCACCAATG-3′; miR-365a-3p: 5′-TGCGGTAATGCCCCTAAAAA-3′ and 5′-TGCAAGAGCAATAAGGATT-3′; U6: 5′-CTCGCTTCGGCAGCACA-3′ and 5′-AACGCTTCACGAATTTGCGT-3′; GAPDH: 5′-CAATGACCCCTTCATTGACC-3′ and 5′-GACAAGCTTCCCGTTCTCAG-3′.

### In vivo study

All animal experiments were approved by the Ethics Committee of Weifang Medical University. A total of 6 × 10^6^ A549 cells were subcutaneously injected into the right flank of the athymic BALB/c nude mice (aged 4 weeks, weight 12–16 g; Vital River, Beijing, China). Once tumors reached about 80 mm^3^, the mice were randomly divided into four groups. Mice were treated with 50 µM/kg erastin by intraperitoneal injection every 2 days for eight times. Tumor size was measured and calculated with the following formula 0.5 × length × width^2^. The tumor tissues, hearts, lungs, spleens, kidneys, and liver were fixed with 4% paraformaldehyde for immunohistochemistry and hematoxylin and eosin staining.

### Immunohistochemistry

Immunohistochemistry for NRF2 was performed on paraffin-embedded xenograft tumor tissue sections. The slides were incubated with primary antibody targeting NRF2 (1 : 200; Abcam, Cambridge, UK) overnight at 4 °C. Slides were incubated with horseradish peroxidase-conjugated anti-rabbit secondary antibody (Protein Tech, IL, USA) for 1 h, then the slides were developed with diaminobenzidine. The images were analyzed by NIH imageJ software.

### Statistical analysis

All data were shown as mean ± SD and analyzed with GraphPad Prism 7 software (La Jolla, CA, USA). Statistical analysis was analyzed by independent *t*-test or one-way analysis of variance test. A *P*-value < 0.05 was considered statistically significant.

## Results

### *MT1DP* sensitizes erastin-induced ferroptosis via repressing of NRF2 in NSCLC

**Fig. 1:**
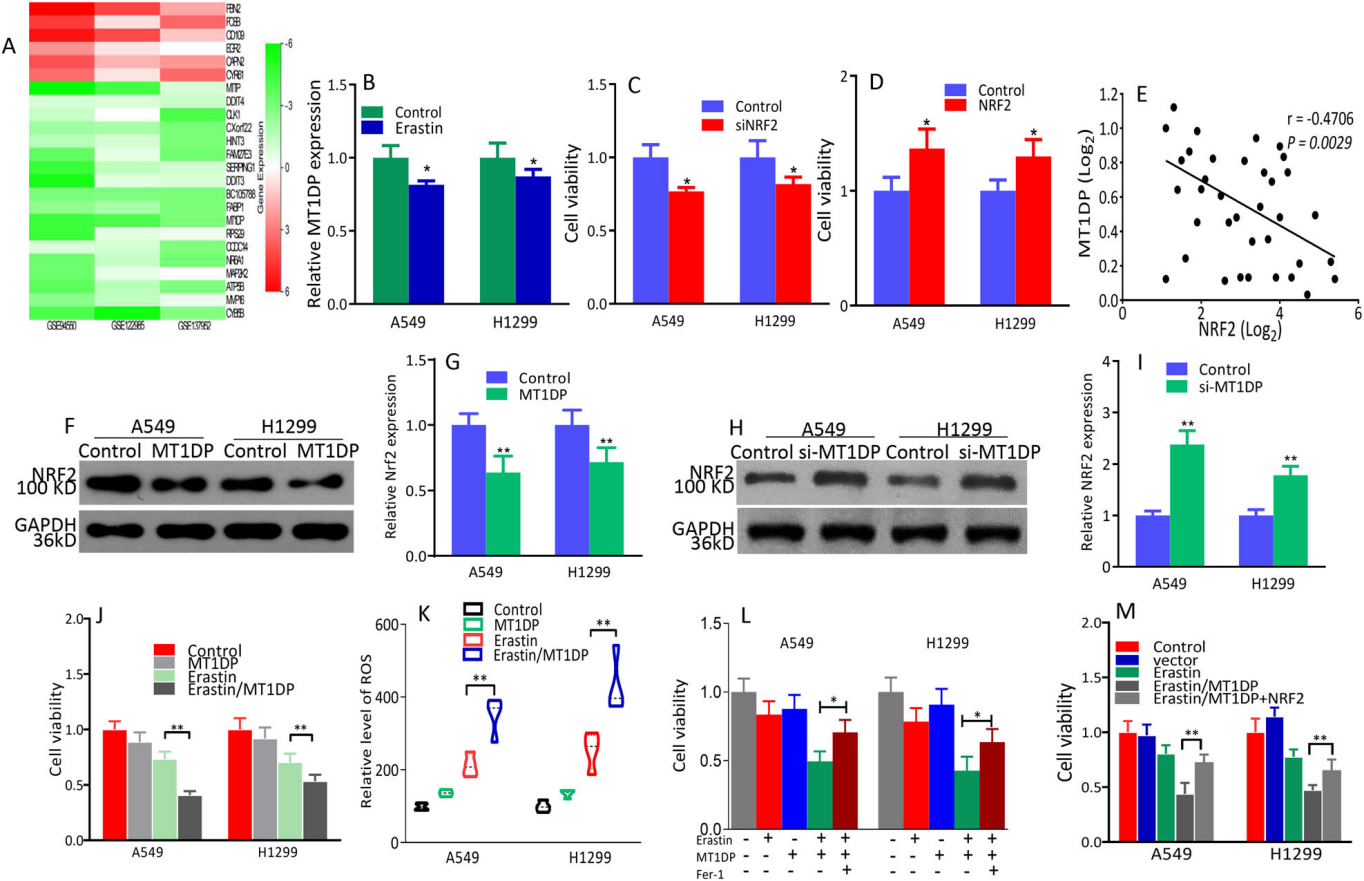
*MT1DP* modulates the expression of NRF2 in NSCLC cells. **a** The heat map shows genes reporting significantly different expression when various malignant tumor cells were treated with or without ferroptosis inducers (i.e., GSE94550, GSE137952, and GSE122985). R package limma was used to identify the differential expressed genes. **b** Expression of *MT1DP* in NSCLC cell lines treated with erastin performed by qPCR. **c** Silencing of NRF2 decreased cell viability. **d** Overexpression of NRF2 enhanced cell viability. **e** Negative correlation between expressions of *MT1DP* and NRF2 in NSCLC samples. **f** NRF2 protein expression was abolished by exogenous *MT1DP* in NSCLC cell lines. **g** NRF2 expression in A549 and H1299 cells with ectopic expression of *MT1DP*. **h** NRF2 were upregulated in A549 and H1299 cells with silence of *MT1DP*. **i** NRF2 mRNA expression by qPCR. **j** Exogenous *MT1DP* rescued erastin-inhibited cell viability of A549 cell. **k** Overgeneration of ROS treated with erastin/*MT1DP* in A549 and H1299 cells. **l** A549 and H1299 cells treatment with Fer‐1 mitigated erastin/*MT1DP*‐induced decrease in cell viability. **m** Exogenous NRF2 in A549 and H1299 cells alleviated reduced cell viability caused by erastin or ectopic *MT1DP*. All experiments were repeated at least three times and representative data are shown. Data are means ± SEM; *p < 0.05, **p < 0.01.

To identify candidate genes that are associated with ferroptosis sensitivity, we performed a bioinformatics analysis from the GEO database (i.e., GSE94550, GSE137952, and GSE122985). Briefly, we screened the data in which clinical drugs (e.g., sulfasalazine, sorafenib, and artesunate) were used to induce ferroptosis, these data displayed that *MT1DP* was one of the significantly downregulated genes in the ferroptosis-insensitive cell lines (Fig. 1a). Our previous studies have demonstrated that A549 and H1299 cells are insensitive to ferroptosis induced by erastin^17^, so we tested the effect of erastin on *MT1DP* expression. Our results found that *MT1DP* expression in NSCLC cells treated with erastin decreased (Fig. 1b). Silencing of NRF2 inhibited cell viability, whereas exogenous NRF2 increased cell viability in both A549 and H1299 cell lines (Fig. 1c, d and Supplementary Fig. S1A, B). A negative correlation between the *MT1DP* expression and NRF2 expression was identified (Fig. 1e). Knockdown or overexpression of NRF2 had no significant effects on *MT1DP* expression (Supplementary Fig. S1C, D). NRF2 expression was downregulated when *MT1DP* was overexpressed in A549 and H1299 cells (Fig. 1f, g and Supplementary Fig. S2A). On the contrary, *MT1DP* silencing elevated the expression of NRF2 (Fig. 1h, i and Supplementary Fig. S2B). A549 and H1299 overexpressing *MT1DP* were more sensitive to erastin compared to the control cells as confirmed by cell viability assay (Fig. 1j); at the same time, ROS was excessively generated (Fig. 1k). The cytotoxicity induced by *MT1DP* in combination with erastin could be relieved in the presence of ferrostatin-1 (Fig. 1l), as shown by MTT. Overexpression of exogenous NRF2 rescued *MT1DP*-mediated sensitivity to erastin (Fig. 1m). These results indicated that *MT1DP* promoted the occurrence of cellular oxidative stress.

### *MT1DP* represses NRF2 via stabilizing miR-365a-3p

**Fig. 2:**
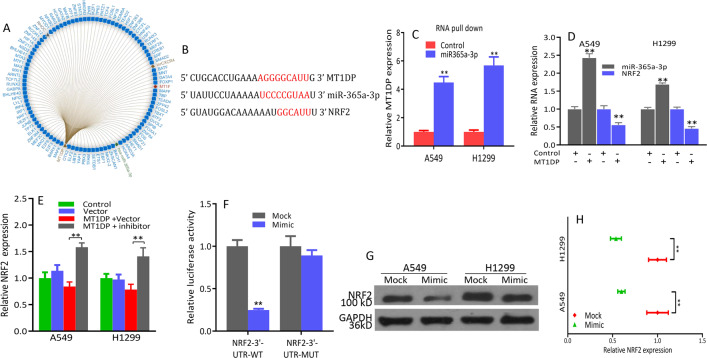
*MT1DP* represses NRF2 via stabilizing miR-365a-3p. **a** The interaction network showed that miR-365a-3p was correlated with *MT1DP* significantly. **b** The binding site of miR-365a-3p within *MT1DP* and 3′-UTR of NRF2. **c** Interaction between miR-365a-3p and *MT1DP* was confirmed by RNA pulldown assay. **d** *MT1DP* overexpression elevated miR-365a-3p and reduced NRF2. **e** *MT1DP*-mediated NRF2 downregulation was abrogated by miR-365a-3p inhibitor. **f** NRF2 was identified as a target of miR-365a-3p by dual-luciferase reporter assay. **g** miR-365a-3p mimic decreased NRF2 expression. **h** miR-365a-3p mimic decreased NRF2 mRNA. All experiments were repeated at least three times and representative data are shown. Data are means ± SEM; **p < 0.01.

RAID v2.0 was used to identify molecules that interacted with *MT1DP*. Indeed, we observed that hsa-miR-365a-3p achieved one of the highest confidence scores among all types of interactors (Fig. 2a); *MT1DP* and NRF2-3′-UTR share a consensus binding site on miR-365a-3p as revealed through bioinformatics prediction (Fig. 2b). We searched for partner molecules that could bind both *MT1DP* and miR-365a-3p, and performed an RNA pulldown experiment using biotin-labeled *MT1DP* and antisense-*MT1DP* as control. As shown in Fig. 2c, miR-365a-3p was more highly enriched in *MT1DP* pulldown cell lysate compared with the control, as shown by quantitative PCR analysis. MiR-365a-3p was enhanced and NRF2 was suppressed in *MT1DP*-overexpressing A549 and H1299 cells relative to control cells (Fig. 2d). MiR-365a-3p inhibitor abrogated the downregulation of NRF2 observed upon *MT1DP* overexpression (Fig. 2e). Dual-luciferase assay was carried out to validate NRF2 as a target of miR-365a-3p; results revealed that miR-365a-3p could suppress the luciferase activity of NRF2-3′-UTR-WT but it had less effect on NRF2-3′-UTR-MUT (Fig. 2f). MiR-365a-3p mimic reduced mRNA and protein expression of NRF2 in A549 and H1299 cells (Fig. 2g, h).

### Characterization of the erastin/*MT1DP* co-loaded FA-LPs

**Fig. 3:**
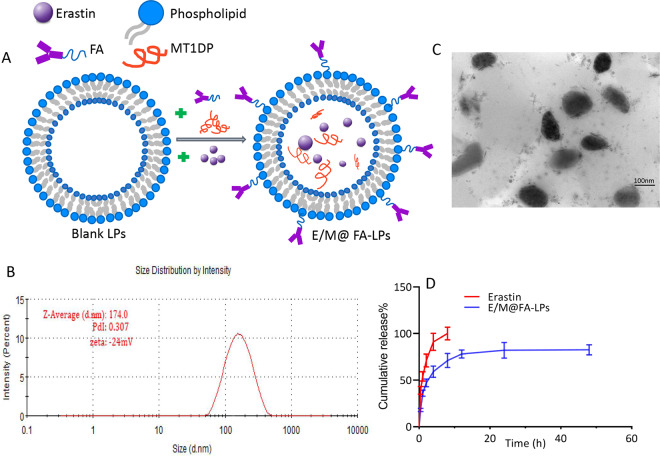
Characterization of E/M@FA-LPs. **a** Schematic diagram of E/M@FA-LPs preparation. **b** The diameter of E/M@FA-LPs performed by DLS is about 174 nm and the zeta potential value was −24 mV. **c** TEM images of E/M@FA-LPs. The micrographs show spherical particles for the E/M@FA-LPs, with the size of 154 nm. Scale bar represents 100 nm. **d** Cumulative erastin release from E/M@FA-LPs. All experiments were repeated at least three times and representative data are shown. Data are means ± SEM.

We loaded erastin and *MT1DP* into FA-labeled liposomes to prepare E/M@FA-LPs (Fig. 3a). The diameter of FA-LPs was 139.7 nm, as shown by dynamic light scattering (DLS) analysis (Supplementary Fig. S3A). The characterization of E/M@FA-LPs by DLS and TEM, showed that E/M@FA-LPs had a size distribution of ~174 nm by DLS, polymer dispersity index of 0.307, and zeta potential was −24 mV (Fig. 3b). As shown in the TEM images, E/M@FA-LPs were confirmed to be of spherical shape and uniform size, and had a particle diameter of about 154 nm (Fig. 3c). The LC and EE of E/M@FA-LPs measured using HPLC are 18.98 ± 2.24% and 48.87 ± 4.83%, respectively. As shown in Fig. 3d, the drug-release profile of erastin reached 95% within the first 8 h; release profile of E/M@FA-LPs had shown initial burst release in the early 2 h followed by a sustained release. Similarly, *MT1DP* is also released from E/M@FA-LPs slowly (Supplementary Fig. S3B).

### E/M@FA-LPs suppress cell malignance

**Fig. 4:**
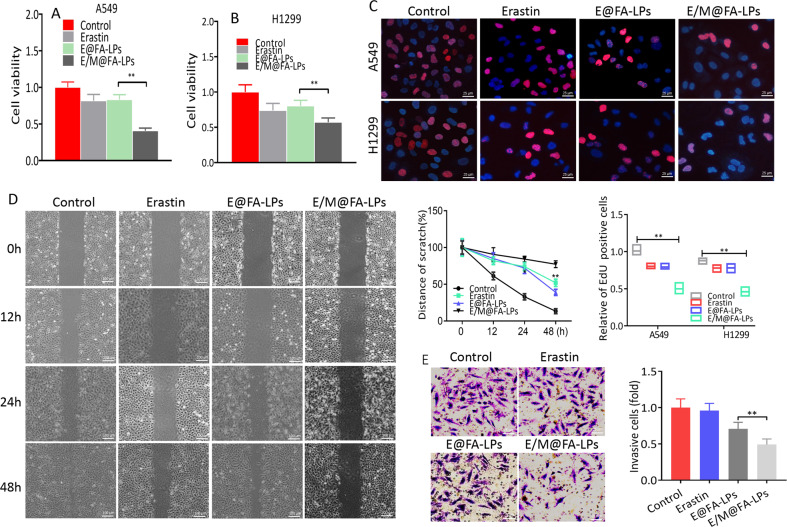
Antitumor effects of E/M@FA-LPs in vitro. **a**, **b** MTT assay showed that E/M@FA-LPs inhibited the viability of A549 and H1299 cells. **c** E/M@FA-LPs inhibited A549 and H1299 cell proliferation by EdU assay. Scale bar represents 25 μm. **d** Effect E/M@FA-LPs on cell migration in A549 cell; the migration of tumor cells to acellular areas is not obvious. Scale bar represents 100 μm. **e** Effect E/M@FA-LPs on cell invasion in A549 cell. Scale bar represents 25 μm. All experiments were repeated at least three times and representative data are shown. Data are means ± SEM; **p < 0.01.

MTT results indicated that E/M@FA-LPs remarkably inhibited cell viability in A549 or H1299 cells compared to E@FA-LPs (Fig. 4a, b). EdU assay was performed to explore the biological effect of E/M@FA-LPs on cell proliferation. As expected, A549 or H1299 cell proliferation was inhibited by E/M@FA-LPs (Fig. 4c). The wound-healing assays demonstrated that the cell migration ability was suppressed significantly upon treatment with E/M@FA-LPs (Fig. 4d). In addition, transwell invasion assay suggested that E/M@FA-LPs significantly reduced the number of invading cells (Fig. 4e).

### E/M@FA-LPs sensitizes erastin-induced ferroptosis in vitro

**Fig. 5:**
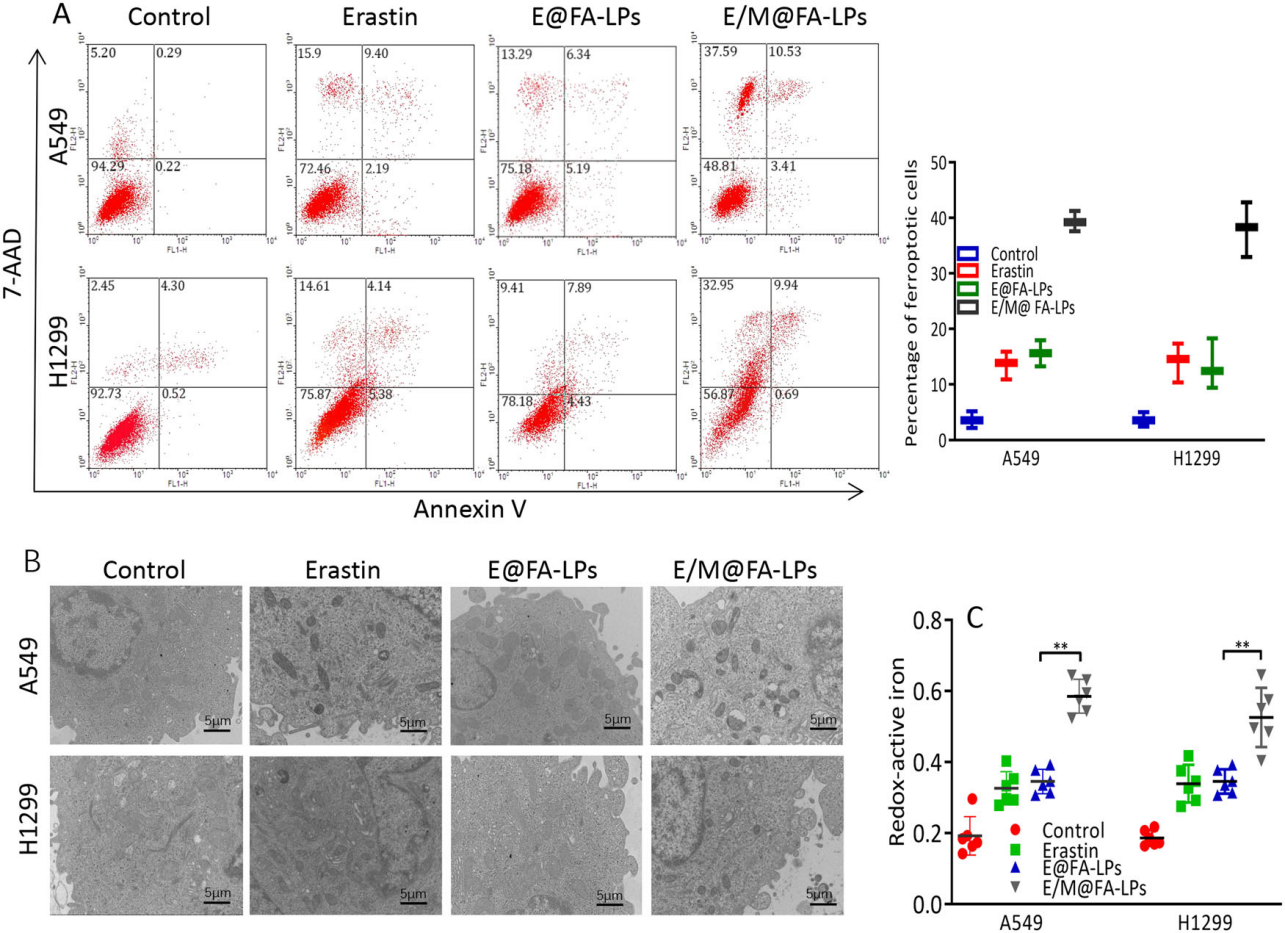
E/M@FA-LPs led to ferroptosis. **a** E/M@FA-LPs contributed to ferroptosis in A549 cell as confirmed by flow cytometry. **b** The A549 and H1299 cells treated with E/M@FA-LPs showed shrunken mitochondria with increased membrane density. Scale bar represents 5 μm. **c** E/M@FA-LPs increased intracellular ferrous iron in A549 and H1299 cells. All experiments were repeated at least three times and representative data are shown. Data are means ± SEM; **p < 0.01.

FCM analysis confirmed that E/M@FA-LPs induced higher levels of ferroptosis compared to those induced by erastin or E@FA-LPs alone (Fig. 5a). Meanwhile, results of TEM brought to light that E/M@FA-LPs contributed to the characteristic changes of ferroptosis on mitochondria with significantly decreased cristae, shrunken mitochondria, and increased membrane density in A549 and H1299 cells (Fig. 5b). Redox-active iron has been implicated as a central player in ferroptosis, so we assessed the iron levels in NSCLC cells treated with E/M@FA-LPs. Intracellular ferrous iron was upregulated significantly in E/M@FA-LPs-treated cells (Fig. 5c).

### E/M@FA-LPs represses NRF2 levels to enhance oxidative stress

**Fig. 6:**
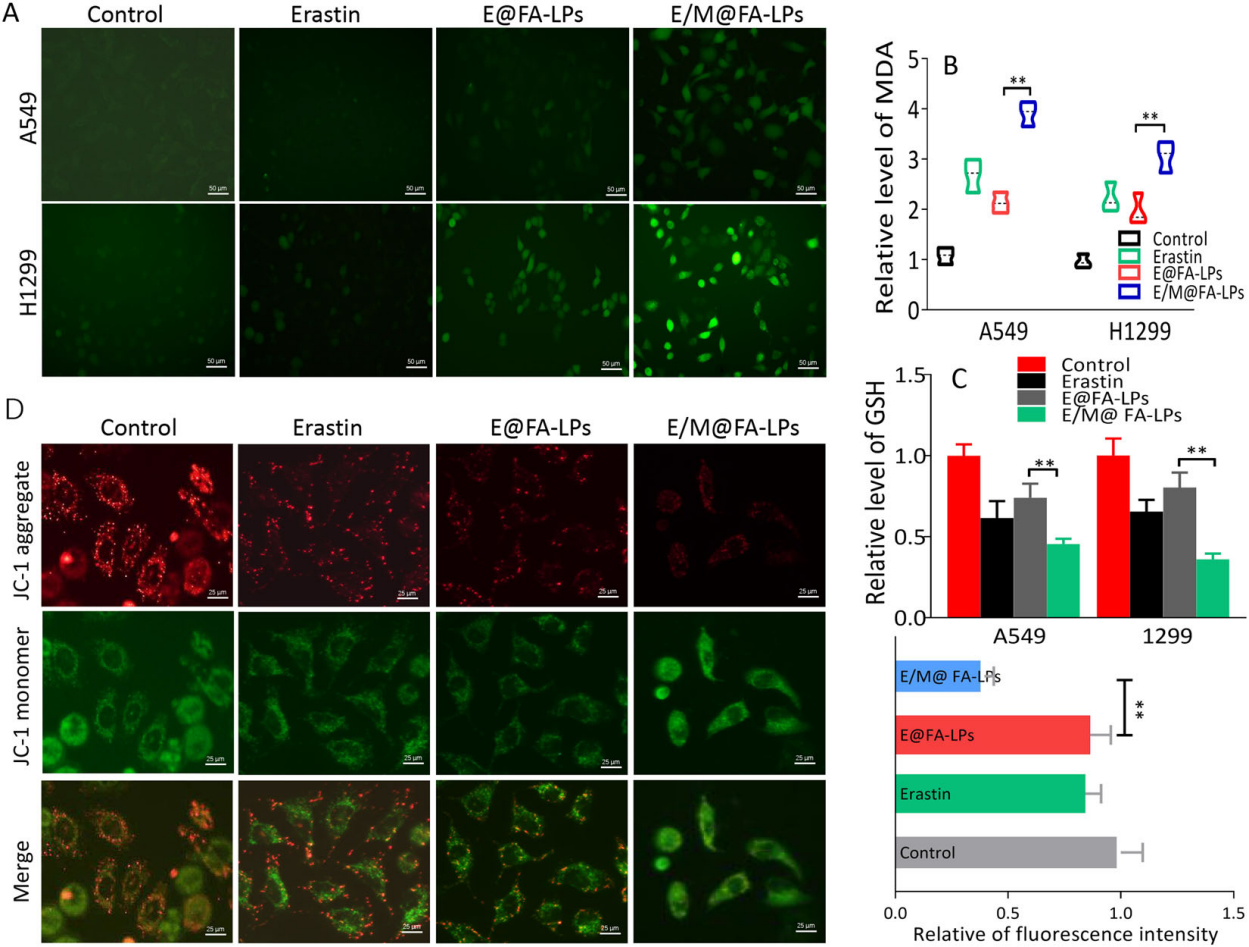
E/M@FA-LPs enhanced oxidative stress. **a** Overgeneration of ROS treated with E/M@FA-LPs in A549 and H1299 cells. Scale bar represents 50 μm. **b** MDA content in different groups of A549 and H1299 cells, E/M@FA-LPs group had the highest MDA overproduction. **c** Intracellular GSH content in different groups of A549 and H1299 cells; A549 and H1299 cells treated with E/M@FA-LPs show the most obvious GSH decline. **d** Mitochondrial membrane potential was detected by JC-1. Scale bar represents 25 μm. All experiments were repeated at least three times and representative data are shown. Data are means ± SEM; **p < 0.01.

ROS, MDA, and GSH were detected to explore the effect of E/M@FA-LPs-induced ferroptosis. ROS and MDA generation were significantly increased in E/M@FA-LPs compared to erastin in A549 and H1299 cells (Fig. 6a, b); in addition, GSH levels were significantly declined by E/M@FA-LPs compared to erastin (Fig. 6c). Previous results suggest that voltage-dependent anion channels play a critical role in the formation of mitochondrial ROS and own potential therapeutic value against glutamate-mediated oxidative toxicity. Here we found that A549 cell treated with E/M@FA-LPs showed higher accumulation of JC-1 monomers and lower accumulation of JC-1 aggregates (Fig. 6d).

### In vivo antitumor effect of E/M@FA-LPs

**Fig. 7:**
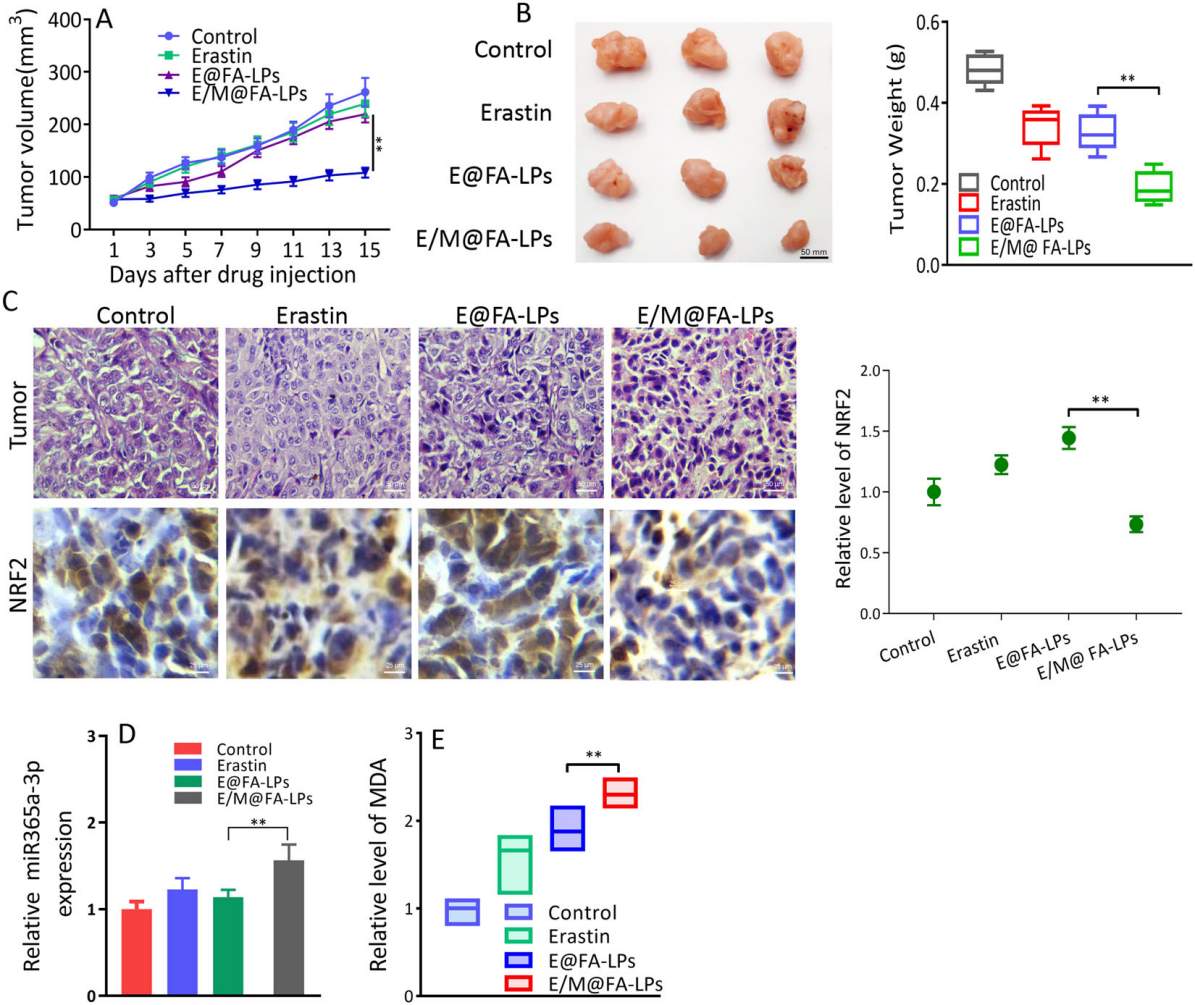
Anti-tumor effects of E/M@FA-LPs in vivo. **a** Powerful growth inhibition of E/M@FA-LPs on subcutaneous xenografts. **b** Weight of xenograft tumors; the weight of xenograft tumors treated with E/M@FA-LPs was lighter. **c** H&E-stained images and immunohistochemistry analysis of NRF2 in xenograft tumors. **d** miR-365a-3p expression in xenograft tumors; miR-365a-3p expression was raised in E/M@FA-LPs group. **e** Overproduction of MDA in xenograft tumors treated with E/M@FA-LPs. All experiments were repeated at least three times and representative data are shown. Data are means ± SEM; **p < 0.01.

E/M@FA-LPs effectively reduced the size of tumors formed by A549 cells compared to E@FA-LP-treated group (Fig. 7a). No significant loss in average body weight occurred (data not shown). The weight of xenograft tumors treated with E/M@FA-LPs was much lighter compared to those from the E@FA-LP-treated group (Fig. 7b). As illustrated in Fig. 7c, more cell death was shown after being treated with E/M@FA-LPs group, whereas only moderate level of cell death was identified in the mice treated with erastin. Tumors were further analyzed by immunohistochemistry, similar to in vitro results, NRF2 expression was decreased in the E/M@FA-LP group (Fig. 7c). MiR-365a-3p expression was augmented in E/M@FA-LP group (Fig. 7d). In addition, E/M@FA-LPs also resulted in more overproduction of MDA (Fig. 7e).

## Discussion

Erastin selectively kills NSCLC cells by inducing ROS accumulation and iron-dependent ferroptosis^2^; unfortunately, its low solubility and metabolic liability preclude its use^4^. Meanwhile, several studies have demonstrated that cancer cells with elevated NRF2 are insensitive to erastin-induced ferroptosis^18^. Therefore, inhibition of the NRF2 signaling pathway could be exploited to induce ferroptosis of NSCLC cells^8,19^.

In this study, we found that downregulation of NRF2 contributed to decrease significantly the viability of A549 and H1299 cells treated with erastin; conversely, overexpression of NRF2 rescued erastin-induced ferroptosis. This might be due to the fact that erastin induces ferroptosis by inhibiting xCT and NRF2 is known to upregulate xCT. Previous findings revealed an important role of *MT1DP* in calibrating the cellular machinery to switch the cellular defense to cytotoxicity through crosslinking with *MT1H*^12^, which plays a crucial role in redox balance^20,21^. Our data clearly demonstrated that exogenous *MT1DP* augmented the effect of erastin on A549 and H1299 cells with excessive production of peroxides. To uncover the mechanism whereby *MT1DP* acts in the cell defense from oxidative stress, we explored the relationship between *MT1DP* and NRF2 in NSCLC samples. We found a negative correlation between the *MT1DP* and NRF2. Exogenous *MT1DP* inhibited NRF2 in A549 and H1299 cells; however, ectopic expression of NRF2 had less effect on regulating *MT1DP*. Overall, these results suggested that NRF2 acts downstream of *MT1DP*.

The interaction between lncRNAs and miRNA contributes to the occurrence of various diseases^22,23^; some lncRNAs work as competing endogenous RNAs to regulate target genes^24,25^. In this study, we found that exogenous *MT1DP* downregulated intracellular miR-365a-3p. The results of an RNA pulldown assay showed that *MT1DP* directly bound miR-365a-3p. Besides, a dual-luciferase assay showed that miR-365a-3p targeted NRF2 mRNA. Moreover, the *MT1DP*-mediated inhibition of NRF2 was rescued by a miR-365a-3p inhibitor, hinting that the regulation of NRF2 by *MT1DP* was miR-365a-3p dependent.

In this work, we further developed FA-LPs to improve the bioavailability of erastin and its therapeutic efficacy towards ferroptosis-insensitive NCSLC cancer by thin-film evaporation technique. E/M@FA-LPs has shown a uniform particle size of 174 nm and a narrow range of distribution, which achieved superior tumor accumulation because of the favorable enhanced permeability and retention effect^26^. E/M@FA-LPs have presented an increased antitumoral efficacy, compared with erastin alone or E@FA-LPs, restraining cell proliferation, migration, and invasion. We found that E/M@FA-LPs promoted ferroptosis of NSCLC cells with excessive amounts of ferrous ion. Treatment with E/M@FA-LPs resulted in increased mitochondrial membrane density and shrunken mitochondria with decreased ΔψM. We discovered that E/M@FA-LPs combined with erastin depressed cell viability and promoted ferroptosis of NSCLC cells; E/M@FA-LPs impaired cell ability to cope with oxidative stress in A549 and H1299 cells, as shown by overproduction of ROS.

**Fig. 8:**
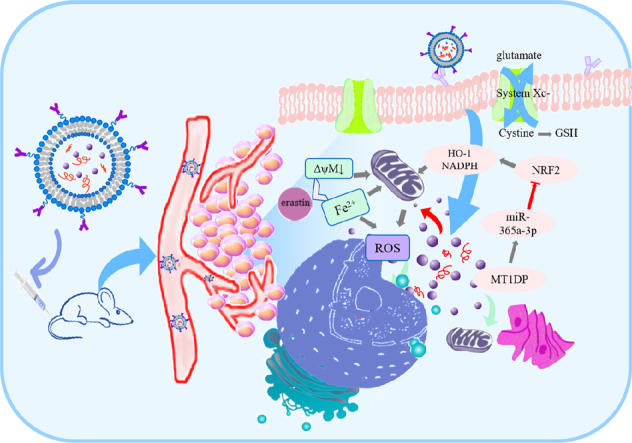
Mechanism of E/M@FA-LPs antitumor in NSCLC xenograft model in mice. Treatment with E/M@FA-LPs led to overgeneration of ROS, ferrous iron and GSH depletion-induced ferroptosis, and depressed growth of xenograft tumors via modulating miR-365a-3p/NRF2 axis.

The role of E/M@FA-LPs-induced ferroptosis was also investigated in vivo. We found that E/M@FA-LPs reduced xenograft tumor volume and weight compared to erastin, which was accompanied by overgeneration of lipid peroxides. E/M@FA-LP-treated xenograft tumors showed lower expression of NRF2 and elevated miR-365a-3p levels (Fig. 8). In conclusion, we propose that *MT1DP* sensitized NSCLC cells to erastin-induced ferroptosis by regulating the miR-365a-3p/NRF2 signaling pathway, and in particular that E/M@FA-LPs might be considered as a successful strategy to sensitize tumors to ferroptosis.

## Supplementary information

Supplementary Figure legend

Figure S1

Figure S2

Figure S3
