# Supplementary material for: MT1DP loaded by folate-modified liposomes sensitizes erastin-induced ferroptosis via regulating miR-365a-3p/NRF2 axis in non-small cell lung cancer cells
Source: Cell Death Dis. 2020 Sep 14;11(9):751. doi: 10.1038/s41419-020-02939-3 (PMC7490417; doi:10.1038/s41419-020-02939-3)
Supplement: Supplementary file 1 — Supplementary Figure legend [file 41419_2020_2939_MOESM1_ESM.docx]

Figure S1 (A) NRF2 expression was abolished by si-NRF2. (B) NRF2 expression of NRF2 was Upregulated by overexpression NRF2. (C) Effects of excessive NRF2 on the expression of *MT1DP* in A549 and H460 cells. (D) Expression of *MT1DP* when NRF2 was silenced in A549 and H460 cells.

Figure S2 (A) *MT1DP* mRNA expression after transfection overexpression *MT1DP* detected by qPCR. (B) *MT1DP* mRNA expression after transfected si-*MT1DP.*

Figure S3 (A) The diameter of E/M@FA-LPs performed by DLS is about 139.7 nm and the zeta potential value was -21 mV. (B) Cumulative *MT1DP* release from E/M@FA-LPs. (C) Exogenous *MT1DP* has no obvious effect on cell viability of A549 and H1299 cell. (D) A549 and H1299 cells treatment with Fer‐1 mitigated E/M@FA-LPs‐induced decrease in cell viability.
