# Supplementary figures and images for: MT1DP loaded by folate-modified liposomes sensitizes erastin-induced ferroptosis via regulating miR-365a-3p/NRF2 axis in non-small cell lung cancer cells
Source: Cell Death Dis. 2020 Sep 14;11(9):751. doi: 10.1038/s41419-020-02939-3 (PMC7490417; doi:10.1038/s41419-020-02939-3)

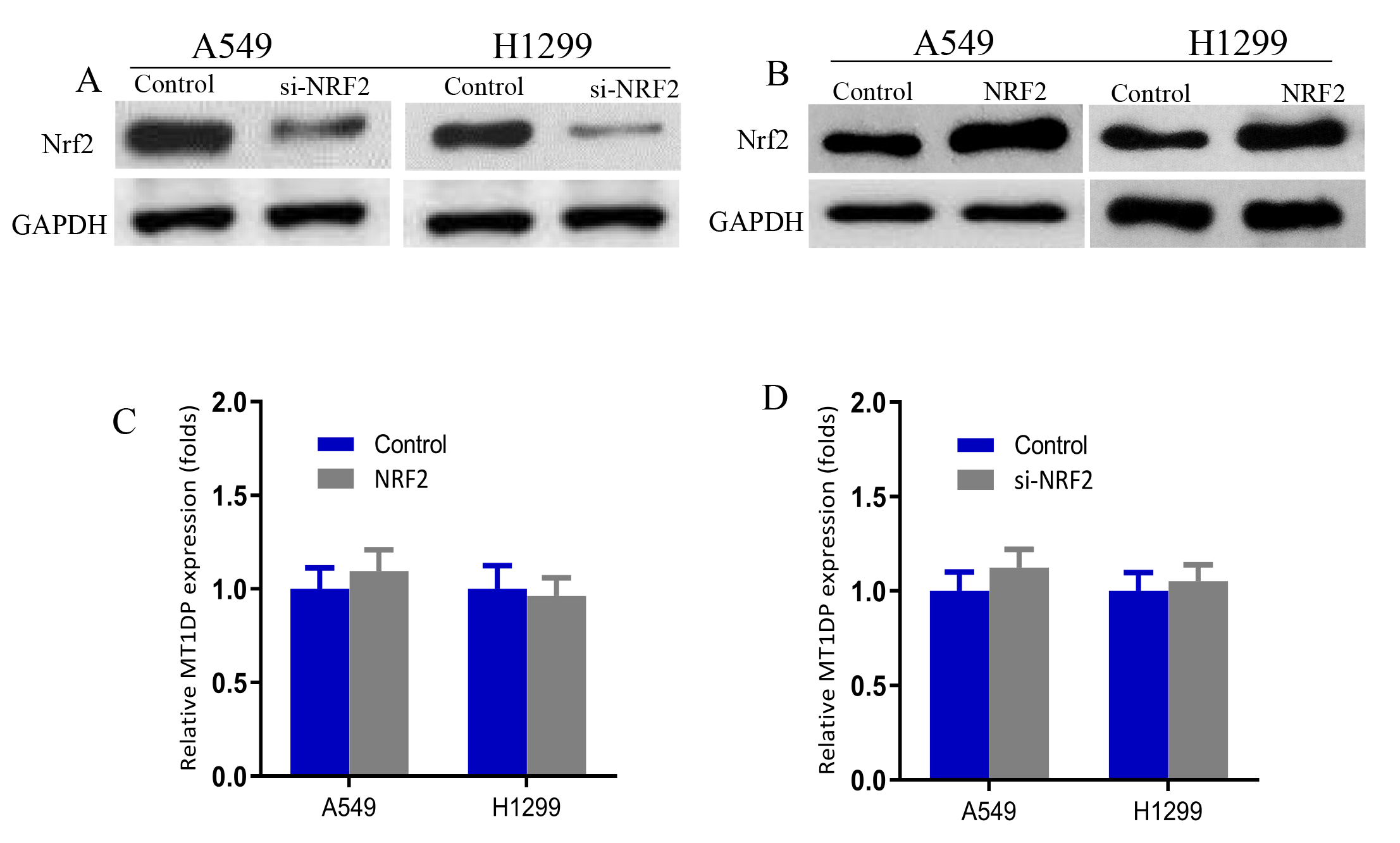

Supplement: Supplementary file 2 — Figure S1 [file 41419_2020_2939_MOESM2_ESM.tif]

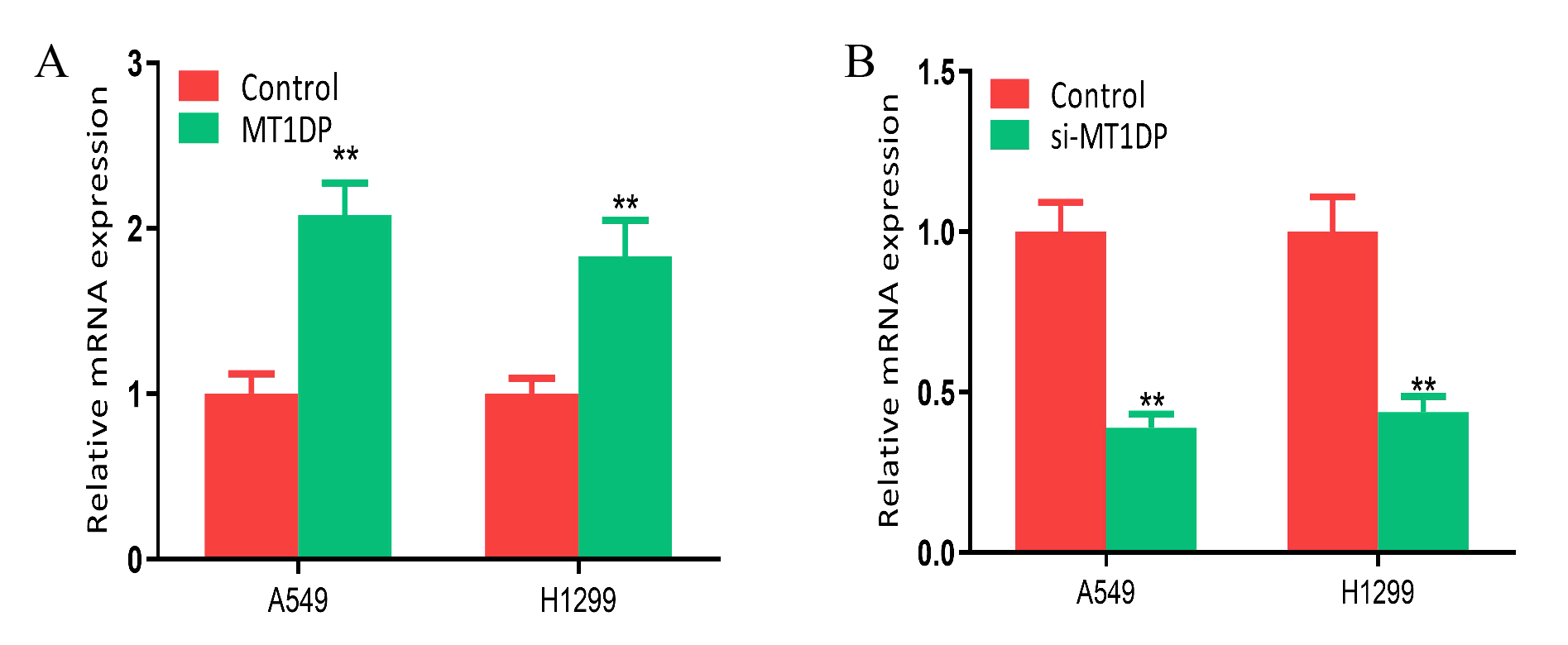

Supplement: Supplementary file 3 — Figure S2 [file 41419_2020_2939_MOESM3_ESM.tif]

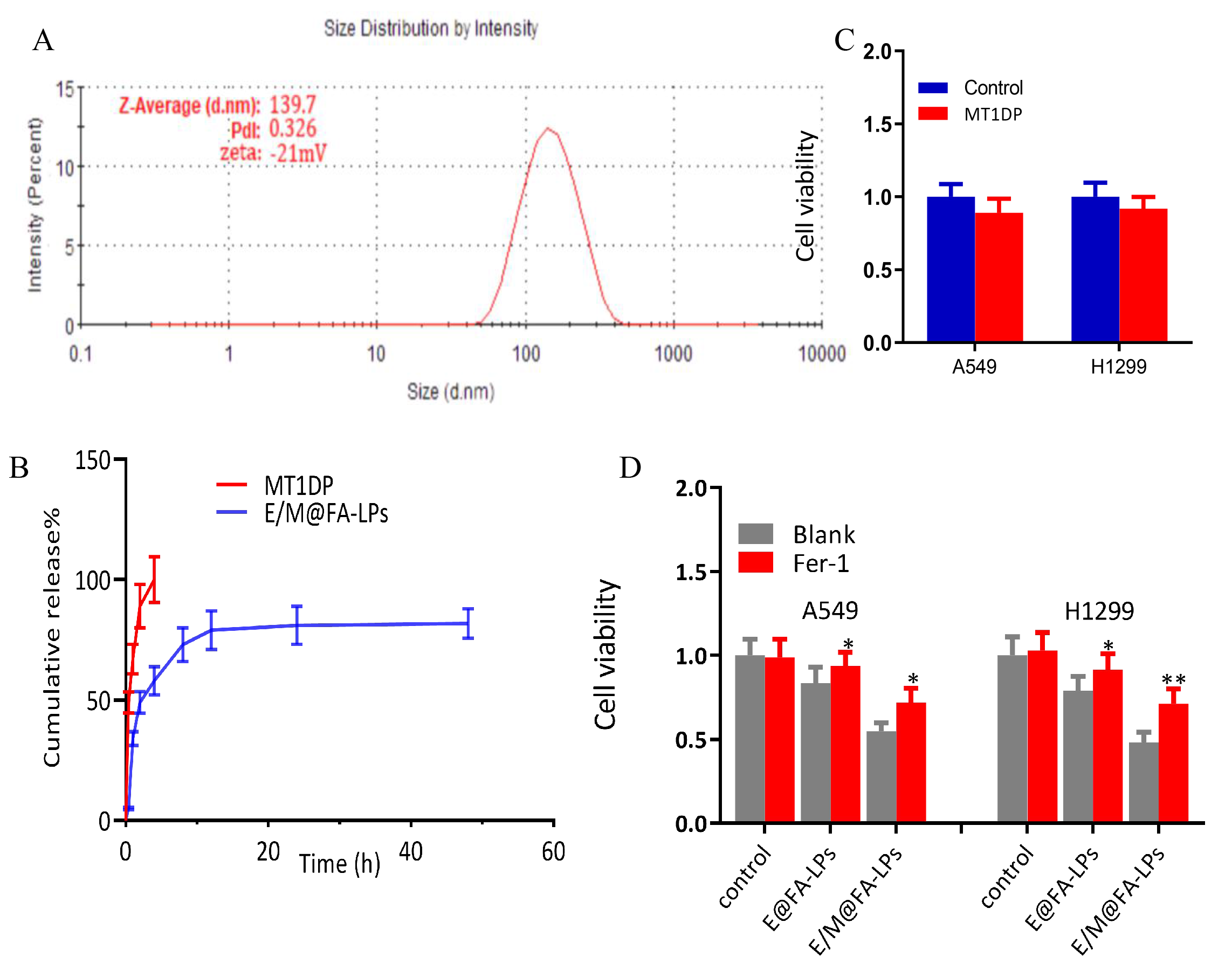

Supplement: Supplementary file 4 — Figure S3 [file 41419_2020_2939_MOESM4_ESM.tif]
